# Tropical Infectious Diseases of Global Significance: Insights and Perspectives

**DOI:** 10.3390/tropicalmed8100462

**Published:** 2023-09-29

**Authors:** André Luis Souza dos Santos

**Affiliations:** Laboratório de Estudos Avançados de Microrganismos Emergentes e Resistentes (LEAMER), Departamento de Microbiologia Geral, Instituto de Microbiologia Paulo de Góes (IMPG), Universidade Federal do Rio de Janeiro (UFRJ), Rio de Janeiro 21941-902, RJ, Brazil; andre@micro.ufrj.br

Neglected tropical diseases (NTDs) are indeed a group of illnesses (Table 1) affecting hundreds of millions of individuals living in tropical and sub-tropical geographical regions of the globe, particularly in socioeconomic vulnerability areas where access to adequate sanitation, a clean water supply, and healthcare is limited [1,2]. In other words, NTDs are intimately associated with poverty and inequity, affecting the most marginalized communities in the world, predominantly living in Africa, Asia, Latin America, and the Caribbean. However, NTDs have the potential to affect anyone, regardless of their geographical location, since climate change can lead to the spread of NTDs to areas that were previously unaffected [3].

NTDs can cause chronic and debilitating illnesses with various consequences, including pain, blindness, disability, disfiguration, reduced productivity, impacts on child development, societal stigma, and, in some cases, death [1,4]. In this context, the World Health Organization (WHO) has recommended five key public health interventions to address the impact of NTDs: (i) preventive chemotherapy (e.g., mass drug administration), (ii) innovative and intensified disease management, (iii) vector ecology and management (e.g., controlling disease-carrying vectors like mosquitoes), (iv) veterinary public health services (e.g., addressing zoonotic diseases), and (v) ensuring access to safe water, sanitation, and hygiene services [1,5,6]. Relevantly, the “One Health” approach, which considers the interconnectedness of human, animal, and environmental health, is definitely recognized as a valuable strategy to combat many NTDs. This approach recognizes that some NTDs can be transmitted between animals and humans and that environmental factors play a role in their transmission. Therefore, addressing NTDs often requires collaboration between human health, animal health, and environmental experts [7].

Relevantly, it is important to highlight that an unstable world has created an uncertain future for the control and elimination of NTDs. However, knowledge is always an excellent tool for change, generating new avenues to improve the management of NTDs. In this context, the present Special Issue published by *Tropical Medicine and Infectious Disease*, titled “Feature Papers in Tropical Medicine and Infectious Disease”, can be considered a viable platform to disseminate new knowledge, insights, and perspectives on NTDs. The present Special Issue is composed of 23 papers (14 research articles, 4 reviews, 3 case reports, and 2 systematic reviews) contemplating the following infectious agents: African trypanosomes (n = 1), amitochondriate protists (1), *Candida* (1), *Cryptococcus* (1), *Helicobacter* (1), influenza virus (1), intestinal protozoan (1), *Leishmania* (4), *Mycobacteium* (1), respiratory syncytial virus (1), Rift Valley fever virus (1), *Saccharomyces* (1), SARS-CoV-2 (3), sexually transmitted microbials (1), *Taenia* (1), *Toxoplasma* (1), *Trypanosoma cruzi* (1), and Zika virus (1). In order to arouse the reader’s curiosity, a brief overview of each published paper is provided below.

The first paper was published by Fatima and colleagues [8], who conducted a systematic review to assess the impact of proton pump inhibitors (PPIs) on COVID-19. The authors concluded that the use of PPI resulted in a significant increase in the risk and severity of COVID-19 infection, resulting in a rise in composite poor outcome and mortality in COVID-19-infected patients. 

Hasan and colleagues [9] published a research paper describing the genetic analysis of influenza A/H1N1pdm strains recovered from patients who were attended at a hospital in Bangladesh in early 2020, immediately before the COVID-19 pandemic.

Tanojo and colleagues [10] suggested that the neutrophil-to-lymphocyte ratio and platelet-to-lymphocyte ratio could be potential biomarkers for the diagnosis of erythema nodosum leprosum, which is an acute immune complex-mediated condition seen in patients with multibacillary leprosy that impacts the patients’ quality of life.

Costa-da-Silva and colleagues [11] presented a review on the role of host immune responses in the establishment, development, and outcome of leishmaniasis, focusing on interactions between innate immune cells and *Leishmania major*.

Roncati and colleagues [12] published a case reported describing left cubital tunnel syndrome in a 28-year-old right-handed healthy male after COVID-19 vaccination. 

Opara [13] described a case report of bilateral cortical blindness caused by neurocysticercosis due to *Taenia solium* in an adolescent Nigerian male. The patient was appropriately treated, resulting in the resolution of most symptoms; however, the patient remained permanently blind. 

Labib & Chigbu [14] reviewed the pathogenesis of ocular manifestations in individuals with Zika virus infection. The authors emphasized immunology, interactions with the host’s immune system, and the pathological mechanisms associated with Zika virus infection.

Guo and colleagues [15] conducted a longitudinal study to analyze the impact of COVID-19 vaccine incentive policies on county-level COVID-19 vaccination rates; the authors also examined the interactive effects between COVID-19 vaccine incentive policies and socioeconomic factors on COVID-19 vaccination rates.

Vasudeva and colleagues [16] conducted a study to evaluate the awareness of sexually transmitted infections (STIs) in men and women from the Sub-Saharan African region, as well as the strength of the association between the awareness of STIs and STIs infection.

Kainga and colleagues [17] conducted a cross-sectional survey using a semi-structured questionnaire to assess the knowledge, attitudes, and management practices regarding Rift Valley fever among livestock farmers in eight districts of Malawi.

Kasozi and colleagues [18] systematically reviewed the factors responsible for the development of African animal trypanocide resistance. The authors stressed that the lack of community knowledge, attitudes, and drug-use practices are the main factors causing the spread of resistance.

Belda and colleagues [19] reported a case of primary cutaneous cryptococcosis caused by *Cryptococcus gatti* in an 80-year-old man. The man’s lesions completely healed after 5 months of treatment with fluconazole.

Ramírez-Soto and colleagues [20] undertook an observational and retrospective study on respiratory illnesses associated with hospitalizations due to the respiratory syncytial virus and influenza virus infections in Peruvian children, comparing the baseline characteristics of sex, age, region, and infection type.

Menezes & Tasca [21] presented a review paper on the potential use of essential oils and terpene-derived compounds as promising chemotherapeutics for drug development against the amitochondriate protists *Entamoeba histolytica*, *Giardia duodenalis*, and *Trichomonas vaginalis*.

Çakırca and colleagues [22] performed a study on the prevalence of toxoplasmosis in pregnant Turkish women. In parallel, the authors correlated this parasitic infection with patients’ general characteristics, clinical and laboratory findings, and pregnancy and fetal outcomes of pregnant women diagnosed with acute toxoplasma infection.

Ramos and colleagues [23] reported a fatal case of sepsis caused by *Saccharomyces cerevisiae* in an elderly Brazilian woman post-infected with SARS-CoV-2. The authors also reported the ability of this opportunistic fungus to produce important virulence factors, which decisively facilitated the establishment of the infectious process in vulnerable individuals.

Wattano and colleagues [24] performed an epidemiological survey on intestinal parasitic infection status, correlating it with socioeconomic status and sanitary conditions among the ethnic minority people of Moken and Orang Laut in Thailand.

Srisuphanunt and colleagues [25] reviewed the molecular mechanisms associated with antimicrobial resistance as well as new and potent treatment strategies for combating infections caused by the Gram-negative bacterium *Helicobacter pylori*.

Silva and colleagues [26] developed a micellar formulation containing 3-carene, a hydrocarbon monoterpene, in order to enhance its anti-*Leishmania* activity using multimodal approaches.

Oliveira and colleagues [27] reported the ability of axenic amastigotes of *Leishmania infantum* to induce human neutrophil extracellular traps (NETs). However, this process did not kill the amastigotes, which, at least in part, justifies the successful infection in vertebrate hosts.

Louise and colleagues [28] demonstrated that theracurmin, a nanoparticle formulation derived from curcumin, was able to modulate cardiac inflammation in an in vivo murine model of *Trypanosoma cruzi* infection.

Garcia and colleagues [29] determined the chemical profile and cytotoxicity of *Allium sativum* essential oil as well as its anti-*Leishmania amazonensis* activity against both promastigote and amastigote forms.

Ramos and colleagues [30] described the aggregation competence, a phenomenon associated with microbial virulence, in clinical isolates belonging to the *Candida haemulonii* clade (*C. auris*, *C. haemulonii*, *C. duobushaemulonii*, and *C. haemulonii* var *vulnera*).

In summary, the present Special Issue offers a collection of high-quality papers in the field of tropical infectious diseases of global significance. The editor truly hopes that the reading of each paper and, of course, the Special Issue as a whole, will arouse enthusiasm and scientific curiosity in young students and researchers around the world. To conclude, the editor would like to take this opportunity to express his gratitude to all contributing authors for their valuable cooperation in adding new information on the proposed theme.

## Figures and Tables

**Table 1:** List of the NTDs recognized by the WHO.

Protozoan NTDs	Helminth NTDs	Viral NTDs	Bacterial NTDs	Fungal NTDs	Other NTDs
African trypanosomiasis (sleeping sickness)	Dracunculiasis (Guinea-worm disease)	Chikungunya	Buruli ulcer	Chromoblastomycoses	Scabies and other ectoparasites
American trypanosomiasis (Chagas disease)	Echinococcosis	Dengue	Leprosy	Deep fungal infections	Snakebite envenoming
Leishmaniasis	Food-borne trematodiases (clonorchiasis, fascioliasis, opisthorchiasis, and paragonimiasis)	Rabies	Trachoma	Mycetoma	
	Lymphatic filariasis	Zika	Yaws		
	Onchocerciasis				
	Schistosomiasis				
	Soil-transmitted helminthiases (ascariasis, ancylostomiasis, and trichuriasis)				
	Taeniasis/Cysticercosis				

## References

[1] World Health Organization (WHO) Neglected Tropical Diseases. https://www.who.int/news-room/questions-and-answers/item/neglected-tropical-diseases.

[2] Engels D., Zhou X.N. (2020). Neglected tropical diseases: An effective global response to local poverty-related disease priorities. Infect. Dis. Poverty.

[3] Tidman R., Abela-Ridder B., de Castañeda R.R. (2021). The impact of climate change on neglected tropical diseases: A systematic review. Trans. R. Soc. Trop. Med. Hyg..

[4] Qian M.B., Chen J., Zhou X.N. (2020). Beating neglected tropical diseases: For good and for all. China CDC Wkly..

[5] Ackley C., Elsheikh M., Zaman S. (2021). Scoping review of neglected tropical disease interventions and health promotion: A framework for successful NTD interventions as evidenced by the literature. PLoS Negl. Trop. Dis..

[6] Nieto-Sanchez C., Hatley D.M., Grijalva M.J., Peeters Grietens K., Bates B.R. (2022). Communication in neglected tropical diseases’ elimination: A scoping review and call for action. PLoS Negl. Trop. Dis..

[7] Peterson J.K., Bakuza J., Standley C.J. (2020). One health and neglected tropical diseases-multisectoral solutions to endemic challenges. Trop. Med. Infect. Dis..

[8] Fatima K., Almas T., Lakhani S., Jahangir A., Ahmed A., Siddiqui A., Rahim A., Qureshi S.A., Arshad Z., Golani S. (2022). The use of proton pump inhibitors and COVID-19: A systematic review and meta-analysis. Trop. Med. Infect. Dis..

[9] Hasan A., Sasaki T., Phadungsombat J., Koketsu R., Rahim R., Ara N., Biswas S.M., Yonezawa R., Nakayama E.E., Rahman M. (2022). Genetic analysis of influenza A/H1N1pdm strains isolated in Bangladesh in early 2020. Trop. Med. Infect. Dis..

[10] Tanojo N., Damayanti A., Utomo B., Ervianti E., Murtiastutik D., Prakoeswa C.R.S., Listiawan M.Y. (2022). Diagnostic value of neutrophil-to-lymphocyte ratio, lymphocyte-to-monocyte ratio, and platelet-to-lymphocyte ratio in the diagnosis of erythema nodosum leprosum: A retrospective study. Trop. Med. Infect. Dis..

[11] Costa-da-Silva A.C., Nascimento D.O., Ferreira J.R.M., Guimarães-Pinto K., Freire-de-Lima L., Morrot A., Decote-Ricardo D., Filardy A.A., Freire-de-Lima C.G. (2022). Immune responses in leishmaniasis: An overview. Trop. Med. Infect. Dis..

[12] Roncati L., Gravina D., Marra C., Della Rosa N., Adani R. (2022). Cubital tunnel syndrome temporally after COVID-19 vaccination. Trop. Med. Infect. Dis..

[13] Opara N.U. (2022). Cortical blindness due to neurocysticercosis in an adolescent patient. Trop. Med. Infect. Dis..

[14] Labib B.A., Chigbu D.I. (2022). Pathogenesis and manifestations of Zika virus-associated ocular diseases. Trop. Med. Infect. Dis..

[15] Guo Y., Gao J., Sims O.T. (2022). Associations between bonus and lottery COVID-19 vaccine incentive policies and increases in COVID-19 vaccination rates: A social epidemiologic analysis. Trop. Med. Infect. Dis..

[16] Vasudeva M., Nakka R., Stock S., Ghebremichael M. (2022). Associations between Awareness of Sexually transmitted infections (STIs) and prevalence of STIs among Sub-Saharan African men and women. Trop. Med. Infect. Dis..

[17] Kainga H., Mponela J., Basikolo L., Phonera M.C., Mpundu P., Munyeme M., Simulundu E., Saasa N. (2022). Assessment of knowledge, attitudes, and practices towards Rift Valley fever among livestock farmers in selected districts of Malawi. Trop. Med. Infect. Dis..

[18] Kasozi K.I., MacLeod E.T., Waiswa C., Mahero M., Ntulume I., Welburn S.C. (2022). Systematic review and meta-analysis on knowledge attitude and practices on african animal trypanocide resistance. Trop. Med. Infect. Dis..

[19] Belda W., Casolato A.T.S., Luppi J.B., Passero L.F.D., Criado P.R. (2022). Primary cutaneous cryptococcosis caused by *Cryptococcus gatti* in an elderly patient. Trop. Med. Infect. Dis..

[20] Ramírez-Soto M.C., Ortega-Cáceres G., Garay-Uribe J. (2022). Characteristics of respiratory syncytial virus versus influenza infection in hospitalized patients of Peru: A retrospective observational study. Trop. Med. Infect. Dis..

[21] Menezes S.A., Tasca T. (2023). Essential oils and terpenic compounds as potential hits for drugs against amitochondriate protists. Trop. Med. Infect. Dis..

[22] Çakırca T.D., Can İ.N., Deniz M., Torun A., Akçabay Ç., Güzelçiçek A. (2023). Toxoplasmosis: A timeless challenge for pregnancy. Trop. Med. Infect. Dis..

[23] Ramos L.S., Mokus L., Frota H.F., Santos M.V., Oliveira S.S.C., Oliveira M.M.E., Costa G.L., Alves A.L., Bernardes-Engemann A.R., Orofino-Costa R. (2023). SARS-CoV-2 post-infection and sepsis by *Saccharomyces cerevisiae*: A fatal case report—Focus on fungal susceptibility and potential virulence attributes. Trop. Med. Infect. Dis..

[24] Wattano S., Kerdpunya K., Keawphanuk P., Hunnangkul S., Loimak S., Tungtrongchitra A., Wongkamchai M., Wongkamchai S. (2023). An Epidemiological survey of intestinal parasitic infection and the socioeconomic status of the ethnic minority people of Moken and Orang Laut. Trop. Med. Infect. Dis..

[25] Srisuphanunt M., Wilairatana P., Kooltheat N., Duangchan T., Katzenmeier G., Rose J.B. (2023). Molecular mechanisms of antibiotic resistance and novel treatment strategies for *Helicobacter pylori* infections. Trop. Med. Infect. Dis..

[26] Silva A.R.S.T., Costa A.M.B., Scher R., Andrade-Neto V.V., Sarmento V.H.V., Santos A.J., Torres-Santos E.C., Jain S., Nunes R.S., Menna-Barreto R.F.S. (2023). Effect of 3-carene and the micellar formulation on *Leishmania* (*Leishmania*) *amazonensis*. Trop. Med. Infect. Dis..

[27] Oliveira T.K.F., Oliveira-Silva J., Linhares-Lacerda L., da Silva Fraga-Junior V., Benjamim C.F., Guimaraes-Costa A.B., Saraiva E.M. (2023). *Leishmania infantum* axenic amastigotes induce human neutrophil extracellular traps and resist NET-mediated killing. Trop. Med. Infect. Dis..

[28] Louise V., Machado B.A.A., Pontes W.M., Menezes T.P., Dias F.C.R., Ervilhas L.O.G., Pinto K.M.C., Talvani A. (2023). Theracurmin modulates cardiac inflammation in experimental model of *Trypanosoma cruzi* infection. Trop. Med. Infect. Dis..

[29] Garcia A.R., Amorim M.M.B., Amaral A.C.F., da Cruz J.D., Vermelho A.B., Nico D., Rodrigues I.A. (2023). Anti-*Leishmania amazonensis* activity, cytotoxic features, and chemical profile of *Allium sativum* (garlic) essential oil. Trop. Med. Infect. Dis..

[30] Ramos L.S., Parra-Giraldo C.M., Branquinha M.H., Santos A.L.S. (2023). Cell aggregation capability of clinical isolates from *Candida auris* and *Candida haemulonii* species complex. Trop. Med. Infect. Dis..

